# Accuracy-Based Glomerular Filtration Rate Assessment by Plasma Iohexol Clearance in Kidney Transplant Donors

**DOI:** 10.3390/jcm12186054

**Published:** 2023-09-19

**Authors:** Zhicheng Jin, Rongrong Huang, Paul Christensen, Roger L. Bertholf, Xin Yi

**Affiliations:** 1 Department of Pathology and Genomic Medicine, Houston Methodist Hospital, Houston, TX 77030, USA; 2 Department of Pathology and Immunology, Baylor College of Medicine, Harris Health System Ben Taub Hospital, Houston, TX 77030, USA; 3 Department of Pathology and Laboratory Medicine, Weill Cornell Medical College, New York, NY 10065, USA

**Keywords:** glomerular filtration rate, plasma iohexol clearance, LC-MS/MS

## Abstract

Background: An accurate measurement of the glomerular filtration rate (GFR) is essential for detecting renal insufficiency in living kidney donors. Iohexol is a “near-ideal” exogenous filtration marker for GFR measurements that has attracted increasing interest in clinical practice because it is non-toxic, non-radioactive, readily available, and easy to measure. In this study, we aimed to set up a laboratory test to conveniently assess the plasma clearance of iohexol in living kidney donors. Methods: A workflow was established in the institution’s infusion clinic to administer iohexol and to collect three timed blood samples from renal transplant donors. Iohexol was thereafter measured by liquid chromatography–tandem mass spectrometry (LC-MS/MS). The serum proteins were precipitated and the supernatant containing iohexol was diluted prior to the LC-MS/MS analysis. The LC-MS/MS method was developed on a Thermo Vanquish UHPLC coupled with a TSQ Endura triple quadruple mass spectrometer with a total run time of 2.5 min. The analytical performance of the method was assessed. Results: The LC-MS/MS method demonstrated a good analytical performance. To calculate the iohexol clearance rate and the GFR, automated data integration and a result calculation were accomplished by using a custom Python script. Automated result reporting was achieved using a laboratory informatics system (LIS) vendor’s direct media interface. Conclusions: We developed and implemented a laboratory test to assess the plasma clearance of iohexol. A workflow was established in the hospital to reliably measure the GFR in living kidney donors, with a potential to be further expanded into other areas where an accurate GFR measurement is needed.

## 1. Introduction

Kidney function tests are important for managing patients with chronic kidney disease (CKD) or an acute kidney injury (AKI). The glomerular filtration rate (GFR), as a property of the kidney, has long been considered the best overall index of kidney function. It is central to defining and staging kidney disease. The GFR cannot be directly measured in humans, but can be evaluated from a clearance measurement of exogenous or endogenous filtration markers, and from GFR-estimating equations. Creatinine clearance and the estimated GFR (eGFR) are the conventional tests used in clinical practice to assess the adequacy of renal function. The calculation of creatinine clearance requires measuring the plasma and urinary creatinine levels and the 24 h urine volume. Creatinine clearance, as a measure of the GFR, is problematic due to the variability in determining an accurate 24 h urinary volume and tubular excretion of creatinine. To overcome these limitations, algorithms have been developed to estimate the GFR based on the plasma level of creatinine or cystatin C.

The chronic kidney disease epidemiology collaboration (CKD-EPI) and the modification of diet in renal disease (MDRD) equations are commonly used algorithms for adults, although other equations have been proposed. The MDRD equation has been derived and validated in patients with an eGFR ≤ 60 mL/min/1.73 m^2^; the CKD-EPI equation provides a more accurate estimate when the eGFR is >60 mL/min/1.73 m^2^ [1]. Although the eGFR provides a more convenient, less expensive, and less invasive approach for GFR assessments in clinical practice, it is less accurate than the measured GFR (mGFR) and has a poor precision for GFR determination in certain circumstances. In addition, both creatinine and cystatin C are influenced by non-GFR factors. The plasma creatinine level is known to be affected by muscle mass and diet, which may lead to interindividual variation in the eGFR. In patients with CKD or type 2 diabetes mellitus, and in renal transplantation recipients, all eGFR equations showed a P30 (the percentage of GFR estimations falling within ±30% of the mGFR values) of less than 90% [2]. Hence, in more than 10% of these patients, the discordance between the eGFR and the mGFR exceeded ±30%. In circumstances where a more accurate assessment of the GFR is needed, such as in living kidney donors, the mGFR is recommended as the confirmatory test.

The measured GFR is considered the most accurate approach to assess the GFR in healthy and diseased populations. Several methods and clearance markers, including inulin, ^51^Cr-EDTA, ^99^Tc-pentetic acid (DTPA), isotopic or non-isotopic iothalamate, and iohexol, have been used to determine the mGFR [3,4]. The renal clearance of inulin is the gold-standard method for GFR determination, but due to its technical complexity, it is not widely available in clinical and research laboratories [5]. Non-isotopic iothalamate and iohexol are non-radioactive markers and are relatively simple to measure in clinical practice and research laboratories. The evidence suggests that the renal clearance of iothalamate and the plasma clearance of iohexol are reliable alternatives to using the inulin renal clearance to measure the GFR [6].

Iohexol is a water-soluble, low-osmolar-contrast medium that is primarily eliminated by glomerular filtration, and it has gained popularity as a reliable marker for determining the mGFR. In assessments of the plasma clearance of iohexol, this agent can be administered as a bolus IV injection, and its plasma concentration decreases according to a two-compartment pharmacokinetic model, comprising a fast component (distribution phase) and a slow component (renal clearance phase). From a plotted curve of the plasma iohexol concentration vs. time, the GFR can be calculated from the area under the curve (AUC) and the dose of the injection. Different protocols have been developed to use single or sparse sampling in the slow component to extrapolate the iohexol clearance curve. From these extrapolated curves, the calculated AUC underestimates the true AUC in the fast-component area, and therefore, mathematical formulae have been developed to correct the underestimation. The correction proposed by Brochner-Mortensen (BM) is the most widely used correction formula [7]. A simple protocol using only two to three different time points together with the BM correction offers accurate and concordant measurements with procedures using multiple sampling points or urinary clearance calculations [3,8]. This protocol was adopted in this study to establish a workflow starting from outpatient infusion clinics to the clinical laboratory to achieve accurate GFR measurements based on the plasma iohexol clearance.

Houston Methodist Hospital is a large transplant center, and therefore, methods to accurately assess the renal function in potential kidney donors are needed. We report the development of an LC-MS/MS method to quantify plasma iohexol and the adoption of a three-point sampling protocol with the BM mathematical correction to assess the mGFR. To facilitate the implementation of this test in our clinical chemistry laboratory services, an interface in the laboratory information system (LIS) was also developed to allow automated calculations, result verification, and reporting. This study is a clinical operation validation study conducted in a clinical laboratory improvement amendments (CLIA) laboratory, and is not considered human subject research. Therefore, it is exempt from IRB oversight and does not require IRB approval.

## 2. Materials and Methods

### 2.1. Reagents

Iohexol (647.1 mg/mL, Omnipaque^TM^ 300 GE Healthcare, Chicago, IL, USA) was obtained from the pharmacy. ^2^H_5_-iohexol was purchased from Santa Cruz Biotechnology (Santa Cruz, CA, USA). Lyphochek drug-free serum was obtained from Bio-Rad Laboratories (Hercules, CA, USA). Methanol (Optima™ LC/MS grade) and formic acid (Optima™ LC/MS grade) were obtained from Fisher Scientific (Hampton, NH, USA). Perchloric acid (70%) was obtained from Sigma-Aldrich (St. Louis, MO, USA). De-ionized water (MilliQ, 18 MΩ, Millipore, Molsheim, France) was made in-house.

### 2.2. Specimen Collection and Storage

The sample collection step occurred in an outpatient setting. Nurses in the infusion clinics were trained in and responsible for administering iohexol, collecting blood samples, and delivering samples to the laboratory after the completion of the whole procedure. Five mL of iohexol was administered to the testing subjects intravenously and blood samples were collected at 120, 180, and 240 min after the administration. The nurses were also asked to record the administered quantity and the exact time of the administration for each sample collected, as well as the patient’s body surface area (BSA). Upon receipt, the samples were centrifuged right away or stored at room temperature prior to centrifugation for up to 24 h. The separated serum or plasma was stored at 4 °C until the analysis.

### 2.3. Standards and Quality Control Preparation

Working solutions with iohexol concentrations of 10 mg/mL and 0.5 mg/mL were prepared by spiking the stock solution (647.1 mg/mL) into drug-free serum. The working solutions were used to prepare calibrators at 5, 10, 50, 250, 750, and 1000 µg/mL in the serum. Another set of working solutions made from a separate stock solution was used to make quality controls at iohexol concentrations of 20, 150, and 800 µg/mL. The calibrators and controls were aliquoted and stored at −20 °C. A ^2^H_5_-iohexol stock solution was prepared by dissolving 1 mg of the standard in 1 mL of water; the standard was aliquoted and stored at −20 °C. The protein precipitation solution containing 1.0 µg/mL ^2^H_5_-iohexol and 2% perchloric acid in water was prepared fresh on the day of the analysis using the ^2^H_5_-iohexol stock solution and 70% perchloric acid.

### 2.4. Sample Preparation

Fifty microliters of a calibrator, control, or serum sample was mixed with 1 mL of the protein precipitation solution and briefly vortexed, followed by centrifugation at room temperature for 10 min at 18,000× *g*. An amount of 100 µL of the supernatant was removed and mixed with 400 µL of water. The injection volume for the LC-MS/MS analysis was 5 µL.

### 2.5. LC-MS/MS Conditions

The LC-MS/MS analysis was performed on a Thermo Fisher Scientific (Waltham, MA, USA) Vanquish UHPLC system coupled with a TSQ Endura triple quadruple mass spectrometer. The LC separation was performed on a Kinetex EVO C18 column (5 µm, 50 × 3.0 mm) with the corresponding guard column (5 µm, 2 × 3.0 mm), operating at 40 °C with a flow rate of 0.6 mL/min. A linear gradient of mobile phase A (0.1% formic acid in water) and mobile phase B (0.1% formic acid in methanol) was programed as follows: 0–0.2 min: 0% B; 0.2–1.2 min: 50–95% B; 1.2–1.7 min: 95–95% B; 1.7–2.0 min: 95–0% B; 2.0–2.5 min: 0% B. The total LC run time was 2.5 min with a total sample acquisition time of 1.5 min. The mass spectrometer was operated in the positive electrospray ionization mode, with a spray voltage of 4.0 kV, an ion transfer tube temperature of 350 °C, a vaporizing temperature of 400 °C, a sheath gas flow rate of 50, an auxiliary gas flow rate of 12, and a sweep gas flow rate of 2. The MS/MS parameters were optimized for each multiple reaction monitoring (MRM) transition, with a universal dwell time of 80 milliseconds. For iohexol, a *m*/*z* of 822.0 to 603.0 was monitored for quantitation with the collision energy at 20, and a *m*/*z* of 822.0 to 804.0 was monitored for confirmation with the collision energy at 25. For ^2^H_5_-iohexol, a *m*/*z* of 827.0 to 809.0 was monitored with the collision energy at 20.

### 2.6. Method Validation

#### 2.6.1. Linearity

The linearity across the calibration range was accessed using linear regression and calculating the coefficient of determination (R^2^). A serial dilution of the 1000 µg/mL standard in serum produced six concentrations from 5 µg/mL to 1000 µg/mL, which were used to verify the linearity of the method.

#### 2.6.2. Lower Limit of Quantification and Limit of Detection

Two aliquots of iohexol-free serum were extracted and injected in duplicate for each of 5 days to obtain the mean and the standard deviation (SD) of the blank signal. The limit of detection (LOD) was defined as the mean of the blank serum signal plus 3 SD. Standards with iohexol concentrations of 0.5, 1, 2, and 5 µg/mL were prepared in the serum. Two replicates of each standard solution were extracted and injected in duplicate for each of 5 days. The between-day precision (CV) was plotted against the concentration. The lower limit of quantification (LOQ) was defined as the concentration corresponding to a CV of 20% or as the mean of the blank serum plus 10 SD, whichever was greater.

#### 2.6.3. Precision and Accuracy

The quality controls at three concentration levels were used to assess the within-run and between-day precision. For the within-run precision, 5 replicates of each QC were extracted and injected 4 times for each replicate. For the between-day precision, 2 replicates of each QC were extracted and injected 2 times for each replicate for 5 days. A CV of ≤15% was considered acceptable. Due to the lack of certified reference material or a higher-order reference method, the trueness of the iohexol measurements could not be assessed. Instead, a method comparison study was performed by splitting the specimens with another lab that has validated a similar LC-MS/MS method to measure the serum iohexol in a clinical setting.

#### 2.6.4. Method Comparison

Iohexol-spiked human serum specimens with iohexol concentrations across the calibration range were prepared and blinded. Aliquots were sent to Ann & Robert H. Lurie Children’s Hospital of Chicago, and the results were compared to those obtained using our method [9]. The correlation data were analyzed using Deming regression and Bland–Altman plots.

#### 2.6.5. Sample Extraction Recovery, Matrix Effect, and Carryover

The iohexol recovery was assessed to evaluate the potential loss during extraction. The iohexol peak areas were compared in samples with the same amount of iohexol spiked in before the extraction vs. samples with the iohexol spiked in after the extraction. The matrix effects were assessed by spiking the same amount of iohexol into blank serum and a blank solvent (DI water) at two different concentrations before the extraction, and comparing the respective iohexol peak areas. The interference from hemolysis, lipemia, and icterus was assessed using the ASSURANCE™ Interference Test Kit (Sun Diagnostics, New Gloucester, ME, USA). The iohexol peak areas were compared between iohexol-spiked serum with and without interference materials added.

A T infusion was used to assess the ion suppression. A blank serum sample after extraction was injected via an autosampler, while 1 µg/mL iohexol in water was continuously infused at a flow rate of 40 µL/min through a syringe pump. Two sides were connected at the T union and infused into the mass spectrometer. The chromatogram was inspected for a potential reduction in the signal at the retention time of iohexol.

The absence of carryover was verified using extracted QC samples and by injecting the low QC in quadruplicate (L1, L2, L3, and L4) immediately after a triplicate injection of the high QC (H1, H2, and H3). The carryover was calculated as [L1 − (L3 + L4)/2]/[(H2 + H3)/2 − (L3 + L4)/2] × 100, and <1% was considered acceptable.

#### 2.6.6. Sample Stability

To assess the iohexol stability in whole blood at room temperature, iohexol was spiked into freshly drawn whole blood, and serum specimens were separated from aliquots of the iohexol-spiked whole blood at 0, 6, and 24 h to measure the iohexol levels. To assess the serum specimen stability, the iohexol-spiked serum specimens were stored at 4 °C and extracted on days 1, 3, and 7 to measure the iohexol levels. To assess the post-extraction stability, the extracted samples were stored at 4 °C and tested on days 1, 3, and 7 to measure the iohexol levels. The freeze–thaw stability of the serum specimens was assessed for up to three cycles. For all studies, a recovery between 85 and 115% was considered acceptable.

### 2.7. Calculation of Normalized GFR

Three serum specimens were collected at 120, 180, and 240 min after a bolus IV injection of iohexol. The iohexol quantity administered and the patient’s BSA were provided on the test order. The iohexol mass spectrometry results file was exported to a custom Python script, which incorporated the test order data to perform the log transformation, linear regression, and calculation of renal clearance (C2 in the BM correction formula) based on the curve extrapolated from the slow component, or the renal clearance phase of a two-compartment model. The GFR was then calculated according to the BM correction, as shown in the equation below [4]:

$$\mathrm{GFR}=0.990778\times C2-0.001218\times C2^{2}$$

The measured GFR was normalized to a BSA of 1.73 m^2^.

## 3. Results

### 3.1. LC-MS/MS Method

**Figure 1:**
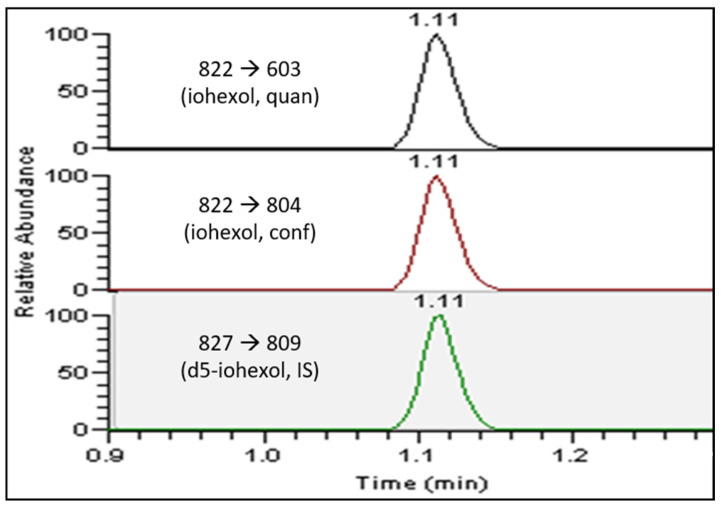
Extracted ion chromatograms of iohexol and d5-iohexol.

Iohexol is a relatively large molecule with a molecular weight of 821.6 Da. Upon collision-activated dissociation (CAD), the base peak was a water loss peak with a *m*/*z* of 804. Commonly, the optimal transition for mass spectrometry-based quantitation was 822 > 804 [9,10]. The additional reported iohexol fragment ions had an *m*/*z* of 603 and an *m*/*z* of 657 [11]. Our study showed that increasing the collision energy increased the relative abundance of the smaller fragment ions. For iohexol, we selected the fragment ion with an *m/z* of 603 as the quantitation ion and the fragment ion with an *m/z* of 804 as the confirmation ion. For the internal standard, ^5^H-iohexol, the peak with an *m/z* of 827 > 809 was chosen for the quantification. As shown in Figure 1, the iohexol peak was eluted at 1.1 min as a single peak.

### 3.2. Linearity, LOD, LLOQ, and Precision

**Figure 2:**
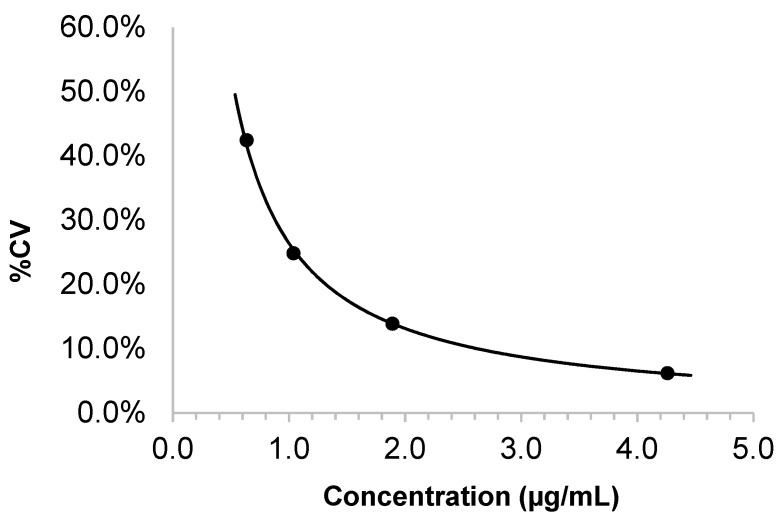
Determination of lower limit of quantitation (LOQ).

The assay was linear from 5.0 to 1000.0 µg/mL with R^2^ > 0.99. Each of the six standards were within ±15% of the targeted concentrations. The mean and SD of the blank (n = 20) were 0.30 µg/mL and 0.28 µg/mL, respectively. The LOD was 1.14 µg/mL. The LOQ was 3.3 µg/mL (S/N of 10) and the corresponding CV at the LOQ was <10%, as shown in Figure 2. The within–run and between–day precisions for each of the three different QC levels are summarized in Table 1; the CVs ranged from 2.7% to 4.4%.

### 3.3. Method Comparison

**Figure 3:**
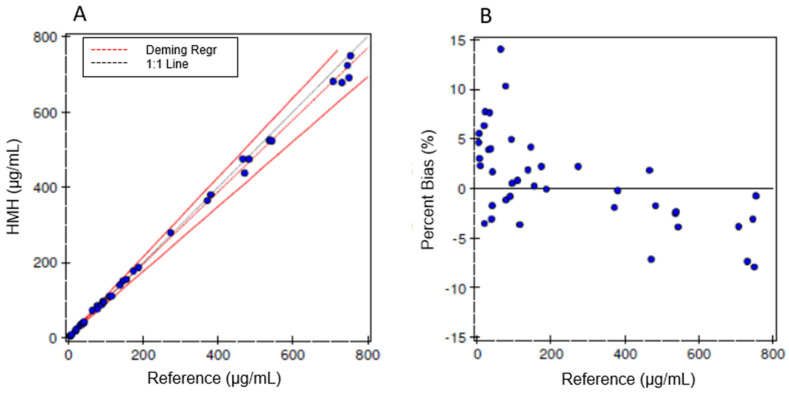
Method comparison study analysis using Deming regression (**A**) and Bland–Altman plots (**B**).

Forty spiked human serum specimens were sent to another hospital laboratory offering a similar iohexol method, and the accuracy of our LC-MS/MS method was evaluated by comparing the two sets of results within the concentration range of 4.3 to 754.3 µg/mL. The correlation data are shown in Figure 3, including the Deming regression analysis and the Bland–Altman plot. The slope was 0.957 (CI: 0.943 to 0.970) and the correlation coefficient was 0.999 (Deming regression).

### 3.4. Additional Validation Summary

The carryover and ion suppression were not significant. The extraction recovery of iohexol was close to 100%, consistent with a lack of significant protein binding. The storage, in-process, and freeze–thaw stabilities of iohexol are shown in Table 2. The method was not affected by hemolysis, lipemia, or icterus at indices of 560, 380, and 50, respectively, as determined by a Roche Cobas analyzer.

### 3.5. Results Interface to LIS

The raw mass spectrometry data containing the specimen barcode numbers and iohexol quantitation results were exported as a comma-separated values (CSV) file directly from the mass spectrometer. The CSV file and test order data in the LIS (dose of iohexol administered, patient body surface area) were integrated using a custom Python script. The script was written by our clinical informatics team to perform the data integration, log transformation, and linear regression; calculate the normalized GFR; and output the results as a final CSV file. The final results were interfaced through our LIS vendor’s (soft computer, SCC) direct media interface.

## 4. Discussion and Conclusions

The iohexol concentrations in the blood collected 2–4 h after the agent injection fell in the range of 10–500 µg/mL, and an LC-MS/MS method with simple pre-treatment steps, such as protein precipitation, provided adequate measurement sensitivity. The measurement accuracy is mainly attributed to the calibration accuracy. Currently, there is no proficiency program for a serum/plasma iohexol test in the United States, but an external quality assessment (EQA) program, Equalis AB (Sweden), offers serum/plasma iohexol proficiency testing and is available for international laboratories. An inter-laboratory comparison study published in 2018 showed a mean difference of <4% for all the participating laboratories except one, which was found to have a greater-than-10% negative bias for their iohexol measurements, leading to a 10–11% positive bias for the measured GFR [12]. If the laboratory results were adjusted using the Equalis program, all the laboratories agreed within 1–2% on the iohexol measurements. Thus, participating in an EQA program or comparing with peer laboratories for iohexol measurements to ensure calibration accuracy is warranted.

In living kidney donor candidates, the 2017 Kidney Disease: Improving Global Outcomes (KDIGO) guideline recommends using an absolute cutoff of a GFR < 60 mL/min per 1.73 m^2^ for exclusion and ≥90 mL/min per 1.73 m^2^ for acceptance, and a GFR of 60 to 89 mL/min per 1.73 m^2^ should be evaluated together with candidates at risk of long-term renal failure based on demographics and other risk factors [13].

In living donor candidates, the eGFR_cr_ based on the CKD-EPI creatinine equation is recommended for the initial assessment of kidney function, and can be confirmed by the mGFR using exogenous filtration markers or the creatinine clearance. Although the eGFR_cr-cys_ or repeated eGFR_cr_ are acceptable alternatives, the most accurate measurement method available in the institution is recommended [13]. The plasma iohexol clearance protocol using a three-point sampling protocol at 120, 180, and 240 min, with an acceptable accuracy and a practical feasibility, was selected in our institution as the confirmatory test for kidney function evaluations in living kidney donor candidates. Iohexol pharmacokinetics are optimally described by a two-compartmental first-order elimination model. The reference plasma iohexol clearance protocol, capturing the full concentration–time decay curve, is inconvenient due to multiple sampling requirements. Simplified alternatives, based on fewer late samples and focusing on the slow compartment of the iohexol clearance curve beyond the 120 min mark, have been suggested [14]. Multiple protocols exist for plasma clearance using single or multiple samples, with different choices of time points. Studies have indicated negligible differences in the GFR values derived from three, four, or five sampling times in the slow compartment, and limited sampling protocols with either three or four draws within a 4 h window have demonstrated an exceptional predictive accuracy in presumptive healthy populations. Other studies have proposed using a one-sample or two-sample protocol to estimate the GFR and have demonstrated a sufficient accuracy and precision [14,15]. However, based on our experience, inaccuracies can arise due to incorrect dosing and imprecise collection time points, both of which can lead to erroneous results. By adding a third time point, such errors can typically be detected in the laboratory by observing a low R^2^ value for the clearance curve, thereby preventing the false reporting of results. Nevertheless, this test may not be ideal in other circumstances. It is suggested by many studies that patients with a low GFR should have an extended protocol with a sampling time of up to 8 h. In more advanced CKD (stage 5) or dialysis patients, an additional sample at up to 24 h has been recommended [7,16]. The plasma iohexol clearance using the short collection protocol (2–4 h) in groups with a GFR < 60 may result in a 25.7–53.3% positive bias compared to the urinary iohexol clearance, or an average of a 7% positive bias in comparison with the GFR measured at up to 8 h [7,16]. However, a long sampling protocol with a late sample at 8 h or at up to 24 h is not easy to implement in clinical practice. Many studies have also proposed single-sample protocols together with different mathematical models to calculate the GFR [17,18,19,20,21]. Clearly, the sampling time of a single-sample protocol is crucial, as well as the precision of the iohexol measurement in the laboratory.

It is not possible to directly measure the “true” GFR in humans, and all methodologies to assess the GFR, either by estimation equations or clearance markers, are associated with imprecisions and errors. As stated above, the inulin clearance with a continuous inulin infusion and urine collection is traditionally regarded as the gold-standard method for measuring the GFR. This method is not only labor-intensive, but has also become increasingly costly in recent times. Alternative clearance markers, including ^51^Cr-EDTA, DTPA, iohexol, and iothalamate, have been employed in both clinical practice and research. These alternatives encompass the renal clearance (based on the marker concentrations in both urine and plasma) and the plasma clearance (based on the marker concentration in plasma). Soveri et al. demonstrated that, when benchmarked against the reference method of inulin urine clearance, the renal clearance of all four exogenous markers exhibited a P30 value greater than 85%. Furthermore, the plasma clearance of ^51^Cr-EDTA and iohexol also achieved a P30 value exceeding 80%. This suggests that these alternative markers and methods offer a satisfactory level of accuracy, making them viable options when accurate GFR measurements are required in a clinical setting [6]. Particularly, numerous studies have compared iohexol with other markers, and have concluded that iothalamate clearance methods demonstrate positive biases (10–15%) for GFR measurements over iohexol clearance methods when using either a plasma or urinary clearance method. A recent study indicated that both the iohexol and iothalamate urinary clearance significantly underestimated the GFR compared to the creatinine clearance, with less bias seen between the iothalamate clearance and the creatinine clearance [11]. However, studies have also suggested a good agreement between the plasma iohexol clearance and the plasma clearance of ^51^Cr-EDTA and ^99^Tc-DTPA [22,23,24]. Even though iohexol is not the perfect exogenous marker for GFR assessments, there are several advantages of using it in a hospital laboratory setting. It is stable in blood, simple to measure, and exhibits low variations between laboratories, making it a reliable and practical method for GFR measurements.

In clinical laboratories across the US, the urine clearance of iothalamate has been the most used alternative method for accurately assessing the GFR. Radiolabeled iothalamate is typically measured using gamma spectrometers. However, the utilization of radioactive agents poses health and safety concerns, particularly when assessing renal donors. For non-radioactive iothalamate, the conventional analytical methods have been HPLC coupled with a UV detector or capillary electrophoresis [25,26]. Before adopting the iohexol protocol, our institution relied on the radioactive iothalamate urine clearance for GFR evaluations in renal donors. This method, in addition to the aforementioned safety concerns, had the potential for an unreliable GFR measurement resulting from inaccurate urine collection. The use of iohexol has gained traction in the US and other countries, attributed to its straightforward single-bolus administration and the need for only a limited number of plasma-collection time points. Our newly developed protocol measures the plasma clearance of iohexol using LC-MS/MS. This approach, which requires minimal sample preparation and boasts a rapid detection time of just 2.5 min per sample run, enhances the accuracy and markedly reduces the analysis time.

The automated result calculation and reporting process used in our laboratory for the plasma iohexol clearance test also streamlined the post-analytical procedures and reduced human errors. With no direct interface available between the mass spectrometry instruments and the LIS, the use of mass-spectrometry-based tests in routine clinical practice has limitations in terms of high-throughput and turn-around time requirements. Alternatively, commercial software or laboratory-developed tools to transfer instrument-generated result text files with a pre-defined format to the LIS via a secure shell connection allows an “indirect” interface between the mass spectrometry instruments and the LIS [27]. Thorough validations of the result file generation, the Python script built for the calculation, and the data transfer to the LIS were performed prior to the implementation of the test. The laboratory technologists were trained and familiarized with the process, and the total time for the technologists to complete the result file generation, result calculation, and verification for one batch was less than 1 min. Overall, the LC-MS/MS method and the post-analytical workflow demonstrated a practical and efficient model to routinely measure the GFR using an exogenous biomarker in living kidney donors, with the potential to further expand to other patient populations.

## Figures and Tables

**Table 1:** Summary of imprecision study.

		QC High	QC Medium	QC Low
Within-Run (N = 20)	Mean (μg/mL)	760.3	133.8	17.9
	CV (%)	4.4%	2.7%	4.1%
Between-Day (N = 20, 10 days)	Mean (μg/mL)	779.0	140.3	18.4

**Table 2:** LDT method validation performance summary.

Specimen type	Serum
Sample storage stability	Up to 7 days of storage at −20 °C and 10 °C
Freeze–thaw stability	Up to 3 freeze–thaw cycles
In-process stability	Stable in vials for up to 7 days when stored at 10 °C
Carryover	Not observed
Extraction recovery	99–101%
Matrix effect	Not observed
Interference (I, L, H) *	Not observed

* I, L, H: icterus, lipemia, and hemolysis.

## Data Availability

Data is unavailable due to privacy or ethical restrictions.

## Abbreviations

GFR: glomerular filtration rate; CKD, chronic kidney disease; AKI, acute kidney injury; CV, coefficient of variation; LOD, limit of detection; LOQ, limit of quantitation; LC-MS/MS, liquid chromatography–tandem mass spectrometry; CAD, collision-activated dissociation.
